# The syndemic challenge of tuberculosis and tobacco use

**DOI:** 10.18332/tid/133575

**Published:** 2021-03-22

**Authors:** Kamran Siddiqi, Thomas E. Novotny

**Affiliations:** 1 Department of Health Sciences, University of York, York, United Kingdom; 2 School of Public Health, San Diego State University, San Diego, United States

**Keywords:** syndemic, tuberculosis, tobacco

The term ‘syndemic’ describes linked health problems involving two or more conditions that act synergistically, causing an excess burden of disease in the population. These interacting conditions often cluster similarly by person, place, and time. Most importantly, interventions against them must address the underlying conditions that bind the problems together^1^. The global incidence of tuberculosis (TB) has declined over the last few decades, albeit far slower than envisioned by the World Health Organization (WHO). For 2018–2019, TB incidence only declined by 2.3%, suggesting that WHO objectives for 2030 will not be met^2^. Ten million persons were infected in 2019, but the burden of TB disease is highest in low- and middle-income countries (LMICs), where 95% of TB deaths occur. The high TB burden countries include India, Indonesia, China, Philippines, and Bangladesh, and these nations also rank among the top ten countries for daily smoking prevalence^3^. Poverty, poor nutrition, and lack of comprehensive healthcare systems in these nations play into the TB and smoking syndemic.

Smoking and secondhand smoke exposure are two of several conditions that exacerbate adverse TB outcomes such as recurrent disease, excess mortality, and treatment failure. These conditions include diabetes, poor nutrition, alcohol use, and HIV infection. Because there are over one billion smokers globally, it is not surprising that 17.6% (95% CI: 8.4–21.4) of new cases and 15.2% (95% CI: 1.8–31.9) of TB deaths are attributable to smoking in high burden countries, regardless of other risk factors^4^.

Given the syndemicity of TB and tobacco use, we might ask what would happen if the smoking prevalence among those infected with TB could be effectively reduced through consistent, integrated treatment for tobacco use. Unfortunately, and despite a higher prevalence of smoking in TB patients compared to the general population^5^, the vast majority of TB patients are neither routinely asked about their smoking status nor advised to quit^6^.

Some progress has been made to meet the syndemic challenge of TB and smoking. There has been increased interest in policies to help TB patients quit smoking^7^ as well as recognition of the need for evidence-based smoking cessation interventions^8^. The WHO and the Union were first to embrace this challenge in the 2007 *Monograph on TB and Tobacco Control,* which called for further research and the application of cessation assistance in TB programs^9^. There are also a few large randomized-controlled trials (RCTs)^8,10^ conducted in high-TB burden countries, which highlight that face-to-face behavioral interventions can achieve high quit rates among TB patients^8^. Those who quit smoking were shown to have better overall clinical outcomes. In these studies, TB staff were able to deliver behavioral interventions for smoking cessation. However, because of several health system barriers^11^ (e.g. cost, reach, sustainability), no high-TB burden country has so far integrated consistent face-to-face behavioral interventions for smoking cessation within its TB services. Recognizing the challenges of integrating and scaling up face-to-face interventions, WHO has developed a smoking cessation package (mTB-Tobacco) that can be delivered as mobile phone messages to TB patients^12^.

The evidence so far points towards a strong syndemic association between adverse TB outcomes and tobacco use. The evidence is also emerging in support of face-to-face behavioral interventions that can be delivered within health services in high-TB burden countries. On the upcoming World TB Day, we want to emphasize the important contribution of tobacco use in sustaining the TB disease burden and to appeal to policymakers and practitioners in high-TB burden countries that they recognize tobacco cessation as an important part of our efforts to end the global TB epidemic.
